# Anxiety, sleep habits and executive function during the COVID-19 pandemic through parents’ perception: a longitudinal study

**DOI:** 10.1186/s41155-023-00251-5

**Published:** 2023-03-29

**Authors:** Ignasi Navarro-Soria, Borja Costa-López, Joshua A. Collado-Valero, Rocío Juárez-Ruiz de Mier, Rocío Lavigne-Cervan

**Affiliations:** 1 Department of Developmental Psychology and Didactics, University of Alicante, Campus San Vicente del Raspeig, 03080 Alicante, Spain; 2 Department of Health Psychology, University of Alicante, Campus San Vicente del Raspeig, 03080 Alicante, Spain; 3 Department of Developmental and Educational Psychology, University of Malaga, Campus de Teatinos S/N. 29071, Malaga, Spain

**Keywords:** COVID-19, Anxiety, Sleep habits, Executive functions, Children, Adolescents, Longitudinal study, Parent report

## Abstract

The present study therefore aims to examine trait and state anxiety, sleep habits and executive functioning during 1 year and a half of the COVID-19 pandemic in children and adolescents through the lens of parents. Assessments were conducted at three different times: April 2020 (T1), October 2020 (T2) and October 2021 (T3). The main sample included 953 children and adolescents aged 6 to 18 years, and scales were used to assess anxiety (STAIC), sleep habits (BEARS) and executive functioning (BRIEF-2). The results showed that 6 months after the outbreak of the pandemic, state and trait anxiety, sleep disturbances and executive dysfunctions increased significantly. One and a half year later, trait anxiety and sleep disturbances have been maintained, while state anxiety and executive dysfunction have decreased their scores obtaining scores similar to those of April 2020. In conclusion, there has been a further decrease in children and adolescents’ mental health since the beginning of the pandemic, and it seems to remain at the present time, such as trait anxiety as a part of the personality.

**Supplementary Information:**

The online version contains supplementary material available at 10.1186/s41155-023-00251-5.

## Introduction

On March 11, 2020, the World Health Organization (WHO) declared a global pandemic caused by COVID-19 (World Health Organization 2020). A couple of days later, the Spanish government imposed a state of alarm, on March 14, 2020. This implied the closure of many workplaces, including primary and secondary schools and also universities (Government Decree 463/2020; Spanish Government, 2020). From then to June 21, 2020, the entire population was forced to a mandatory lockdown. At the time, this was an unexpected event. Thus, no previous research studies could initially help to predict the psychological impact of such unusual circumstances. However, some studies developed in relatively similar smaller-scale contexts, such as more local epidemics, seriously warned of the possible impact of prolonged isolation on mental health, mainly in children and adolescents (Han & Lee, 2018; Hoven et al., 2005; Laor et al., 1997; Park et al., 2020). Therefore, the scientific community was focused not only on the physical health of people infected with COVID-19 but also on the psychological impact on vulnerable groups, such as children and adolescents (Jiloha, 2020).

Lavigne et al. (2021a) suggested through a path analysis that there was a strong correlation between state/trait anxiety, sleep disturbances and executive functioning during COVID-19 confinement.

Other recent studies carried out in Spain and other countries examined the perception of parents about their own mental health and that of their children. In fact, these studies showed that the stressful situation maintained by the pandemic triggered high levels of emotional and behavioural problems, alterations in the sleeping and eating habits and also a decrease in the expression of positive effects, worsening the mental health of families (Andrés et al., 2022; Brown et al., 2020; Watkins-Martins et al., 2021; Romero et al., 2020).

Most scientific evidence that has associated the COVID-19 pandemic with mental health in the child and adolescent population comes from cross-sectional studies conducted during the original COVID-19 confinement or the first wave of coronavirus — from March to April, 2020 (Elharake et al., 2022). In fact, many articles agree that anxiety was one of the main variables, due to its close relationship with traumatic events (Collimore et al., 2010). Nevertheless, the results of such research differ depending on their developmental context. For instance, several studies have found the presence of elevated rates of anxiety in 20% of children and adolescents in China (Zhang et al., 2021), Brazil (Garcia de Avila et al., 2020), Germany (Ravens-Sieberer et al., 2021) and Bangladesh (Yeasmin et al., 2020). Other reports have estimated proportions of 30% in Italy (Pisano et al., 2020), 50% in the UK (Levita et al., 2020) and Egypt (El Refay et al., 2021), 60% in Spain (Lavigne-Cerván et al., 2021) and 70% in India (Saurabh & Ranjan, 2020).

On the other hand, recent authors have hypothesised and demonstrated the direct impact of COVID-19 confinement on sleep quality (Lavigne-Cerván et al., 2021a). Research conducted on sleep characteristics in particular demonstrated it unstructured. These indicated the delay of sleep routines, with both bedtime and wake-up times being delayed (Kaditis et al., 2021), as well as the prolongation of rest time (Liu et al., 2021). However, it is important to consider that more hours in bed at night may not necessarily imply a good quality of sleep. Therefore, these results could be similar to other investigations that find from a deterioration of rest (Cellini et al., 2020) to alterations such as insomnia, nightmares and night terrors (Zhou et al., 2020).

Moreover, high levels of anxiety present a positive correlation with sleep disturbances (Cox & Olatunji, 2016). Thus, a large number of researchers has also assessed the direct impact of the first wave of coronavirus on sleep hygiene in children and adolescents, revealing changes in sleep patterns and a deterioration in sleep quality (Becker et al., 2021; Cellini et al., 2020; Kaditis et al., 2021; Zhou et al., 2020). The most powerful meta-analyses hence estimated that between 40 and 50% of the child and adolescent population reported sleep problems during the initial COVID-19 lockdown (Jahrami et al., 2022; Sharma et al., 2021).

Similarly, a slight amount of studies have observed that some cognitive parameters such as executive functioning have also been impaired (Hanno et al., 2022). Lavigne-Cervan et al. (2021b) evidenced more prevalence of anxiety and poor executive functioning in children and adolescents during the online learning situation resulting from the pandemic. Individuals suffering from high levels of anxiety tended to present poorer emotional management, cognitive rigidity and disorganisation. Moreover, both elevated rates of anxiety and sleep disturbance were positively correlated with impaired executive functioning (Tham et al., 2017; Weyandt et al., 2014). On the other hand, Polizi et al. (2021) suggested that the most distressed parents perceived their children as less competent in executive functioning, highlighting a cognitive fragility of attention, memory and self-regulation. Those few studies have found a significant deficit in the components of executive functioning, although they have stated an adequate executive functioning could work as a protective factor for mental health in situations of traumatic stress (Green et al., 2021; Ji & Saylor, 2021; Ji et al., 2021; Kira et al., 2021).

Potential consequences arising from COVID-19 restrictions on the mental health of children and adolescents require to be reconsidered with a long-term perspective (Wade et al., 2020). Research evidence regarding the relationship between the COVID-19 pandemic and the mental health of the child and adolescent population of longitudinal studies is less prevalent compared to cross-sectional ones (Elharake et al., 2022). Some of the studies that took measurements in the first month following the initial coronavirus wave (from May to August, 2020) found apparent reductions in youth and adult mental health, stress, anxiety and depression (Fancourt et al., 2021; Watkins-Martin et al., 2021a, 2021b). Several researches have indicated a partial decrease in mental health problems in the early summer of 2020 (Robinson et al., 2022). Other studies observed significant decline in mental health problems by the end of summer 2020, achieving similar rates to those observed prior to the COVID-19 pandemic (Daly & Robinson, 2021). Notwithstanding, the arrival of the second wave of the coronavirus (from September to December, 2020) brought an increase in emotional alterations (Falkingham et al., 2022). Some studies found that the rise of mental health problems, which began in autumn and persisted until winter, progressively increased and even exceeded those found at the beginning of the pandemic (Giménez-Dasí et al., 2021; Vahratian, 2021).

Some researchers observed a greater psychological impact, compared to the emotional deterioration of March 2020, which remained stable from December 2020 to February 2021 (Giménez-Dasí et al., 2021; Vahratian, 2021). One year after the outbreak of the COVID-19 pandemic, other research works also showed an increase in the level of distress above the scores collected during the first lockdown resulting from this pandemic (Ausín et al., 2022). In addition, another longitudinal study comparing measures taken in March 2020 and 2021 revealed a deterioration in mental health, even at lower levels during the first wave of coronavirus (Ettman et al., 2022).

For all these reasons, there is no doubt that from March 2020 to November 2021, everyone’s life changed. Despite the fact that previous research with cross-sectional designs have reflected the impact of confinement and the COVID-19 pandemic on children and adolescents’ mental health, to date, there is a scarcity of studies assessing how the psychological variables have changed over the past 2 years. The present study therefore has the main objective of analysing the evolution of some psychological variables in children and adolescents residing in Spain (state and trait anxiety, sleep disturbances, executive functioning-emotional dysregulation, cognitive rigidity and disorganisation), using three time points of reference during the COVID-19 pandemic with different levels of restriction, according to parents’ perception.

Specifically, this general objective is divided in four specific objectives: (1) to examine parents’ perceptions regarding the level of state and trait anxiety, sleep disturbances, executive functioning as a general index, emotional dysregulation, cognitive rigidity and disorganisation in Spanish children and adolescents at three time points (T1, T2, T3); (2) to determine whether there are significant differences between the three time periods (T1, T2, T3) in the variables measured in the same sample; (3) to test the relationship of anxiety, sleep habits and executive function during the COVID-19 pandemic in different restriction contexts (T1, T2, T3); and (4) to analyse the influence of the anxiety and sleep habits on the executive functions in the study sample during the COVID-19 pandemic in these different restriction contexts (T1, T2, T3).

## Method

### Research design, study context and participants

The present study followed a longitudinal design, based on a methodology of observational research. This research was conducted online in three different times (T1, T2, T3) with children and adolescents from different Spanish cities. Children’s mean age was 10.73 (*SD* = 3.24), and 57.11% were males. The inclusion criteria for the entire sample were as follows: (a) 6 to 18 years of age and (b) residence in Spain during the development of the study.

The first sample (T1) included 953 children and adolescents, who were in quarantine due to the COVID-19 pandemic in April 2020. Participants for the first time were contacted to be recruited for the second time, and 134 children and adolescents participated in October 2020. The same procedure was carried out for the third sample in October 2021, and 53 youngsters finally collaborated in the study. The last two samples were not in quarantine.

Considering the possibility of the presence of biases in the obtained results related to the loss of sample through time, difference analyses were taken into account. Compared to responders at time 2, T1 nonresponders for the T2 were slightly younger (0.28 years difference, *p* = 0.351) and hardly ever less females (1.38% difference, *p* = 0.728). For baseline measures, there was no difference between responders and nonresponders in trait (*p* = 0.64) and state anxiety (*p* = 0.09), sleep disturbances (*p* = 0.32) and executive functioning (*p* = 0.275) at T2.

Compared to responders at time 3, T2 nonresponders for T3 were slightly younger (0.14 years difference, *p* = 0.794) and hardly ever less females (7.04% difference, *p* = 0.205). For baseline measures, there was no difference between responders and nonresponders in trait (*p* = 0.695) and state anxiety (*p* = 0.404), sleep disturbances (*p* = 0.728) and executive functioning (*p* = 0.349) at T3.

Moreover, the participation of parents was required to report children and adolescents’ information in relation to what was observed during the COVID-19 pandemic. Regarding their sociodemographic data, most of them were females, and they were in a relationship, with a mean age of 43.95 (*SD* = 6.54). A higher proportion of parents held an undergraduate level at three times, and most of the parents also received more than 950 € as a monthly income. All participants appeared to be able to understand the Spanish language. Parents with sensory, physical or psychological deficits that made it difficult for the participant to complete the evaluation instrument were excluded.

Although a large number of participants decreased in T2 and T3, the obtained sample was considered valid, since the research tried to observe these children and adolescents during the three periods of time. Furthermore, this methodology maintained similar sample’s characteristics among the three different times. Sociodemographic data of the samples are shown in Table 1.

**Table 1:** Sociodemographic data of the samples

	T1 (*N* = 953)	T2 (*N* = 134)	T3 (*N* = 53)
Parents			
Females, *n* (%)	804 (84.37)	114 (85.08)	46 (86.79)
Age, M (SD)	43.30 (6.70)	44.17 (6.21)	44.38 (6.70)
Relationship status, *n* (%)			
In a relationship	794 (83.32)	117 (87.31)	46 (86.79)
Single	159 (16.68)	17 (12.69)	7 (13.21)
Educational level, *n* (%)			
PhD/master’s degree	128 (13.43)	23 (17.16)	13 (24.53)
Undergraduate	401 (42.08)	69 (51.49)	28 (52.83)
Secondary school	383 (40.17)	39 (29.11)	12 (22.64)
Primary school	41 (4.30)	3 (2.24)	-
Monthly family income, *n* (%)			
Up to MIW^a^	219 (22.98)	15 (11.19)	5 (9.43)
MIW^a^	141 (14.80)	15 (11.19)	4 (7.55)
More than MIW^a^	593 (62.22)	104 (77.61)	44 (83.02)
Children			
Females, *n* (%)	439 (46.16)	60 (44.78)	20 (37.74)
Age, M (SD)	10.86 (3.29)	10.61 (3.14)	10.72 (3.30)

^a^Minimum interprofessional wage (MIW), established by the Spanish government. In 2020, the MIW was 950 €. In 2021, the MIW was 965 €

### Instruments

#### Structure of the questionnaire

A tool was designed to be filled out by parents or legal guardians of the participants. The time of fulfilment required was 15–20 min, roughly. This instrument was structured in four sections. The first section was prepared from an ad hoc sociodemographic questionnaire related to the contextual and psychosocial characteristics of the families (relationship status, educational level and monthly family income) given the situation at each moment (T1, T2, T3), age and sex. The subsequent sections gathered information from different standardised scales, whose items were used and adapted to be completed by adult observers.

#### State and trait anxiety in children and adolescents

The State-Trait Anxiety Inventory for Children (STAIC; Spielberger et al., 1973) was based on the State-Trait Anxiety Inventory (STAI; Spielberger et al., 1971), which was created to assess both state and trait anxiety. The STAIC has two independent scales comprising 10 items each. This instrument originally demonstrated good psychometric properties and a high reliability through the Cronbach alpha’s internal consistency coefficient ranged from *α* = 0.78 to *α* = 0.81 for the Trait Anxiety scale and from *α* = 0.82 to *α* = 0.87 for the State Anxiety scale (Spielberger et al., 2015). Each item provides 3 response options (1 = not at all, 2 = somewhat, 3 = very much); however, the current study has modified the rating response scale to a 7-point Likert-type scale with higher scores indicating greater anxiety. Furthermore, despite the original application of the instrument was self-administered, parents completed the questionnaire in this research. Therefore, items were changed. Example items include the following: “My child is feeling calm”, instead of “I am feeling calm”. This instrument was also validated, standardised and normalised in the Spanish population (Spielberger et al., 2015). The reliability for the present study was good, with a Cronbach’s alpha of 0.89 for trait anxiety and a Cronbach’s alpha of 0.91 for state anxiety. According to the meta-analysis developed by Guillén-Riquelme and Buela-Casal (2015), the STAI has high reliability indicators (*α*-values of 0.87 and 0.93). A more recent study (Ortuño-Sierra et al., 2016) corroborates the internal consistency (*α*-values between 0.94 and 0.98) and stability of the STAI (*r*-values between 0.81 and 0.93) in general and clinical populations.

#### Sleep disturbances

The Sleep-Screening Tool for Sleep Disturbances in Childhood (BEARS; Owens and Dalzell, 2005; Ramírez-Vélez et al., 2018), the BEARS, is a 9-item test used to detect sleep disturbances in children and adolescents between 2 and 18 years of age. The BEARS is considered a reliable measure of sleep disturbances for children and adolescents (Owens and Dalzell, 2005; Bastida-Pozuelo & Sanchez-Ortuno, 2016; Ramírez-Vélez et al., 2018). The BEARS has 5 subscales, and all of them were used for analyses in this study: bed-time problems, excessive daytime sleepiness, awakenings during the night, regularity and duration of sleep and snoring. This test was completed by parents or legal guardians answering questions such as “Does your child have trouble going to bed?” or “Does your child have trouble waking up in the morning?”. Although the original version used a dichotomous response, in the current study, items were scored on a 7-point Likert-type scale (1 = totally disagree and 7 = totally agree). The BEARS was also validated in the Spanish population (Bastida-Pozuelo & Sanchez-Ortuno, 2016). The scale demonstrated good reliability and internal consistency for this study, with a Cronbach’s alpha of 0.732.

#### Executive functioning

The Behavioral Evaluation of Executive Function-2 (BRIEF-2; Gioia et al., 2015). The BRIEF-2 is a 63-item measure of executive behaviour in children between 5 and 18 years of age. The BRIEF-2 has a family version, which can be completed by parents or other relatives. The original version consists of 9 scales with items rated on a 3-point Likert-type scale (always, sometimes or never). Higher scores indicate poorer executive functioning. Nevertheless, the present study used a 12-item test with a general index of executive functioning and three scales: 6-item emotional regulation, 4-item cognitive flexibility and 2-item organisation/planification. Furthermore, the rating response scale was modified to a 7-point Likert-type scale. The BRIEF was also validated, standardised and normalised in the Spanish population (Gioia et al., 2017). The scale demonstrated acceptable reliability with well-defined discrimination indexes ranging from 0.46 to 0.72 in emotional regulation, from 0.29 to 0.37 in cognitive flexibility and 0.56 in organisation/planification.

### Procedure

This study was approved by an institutional ethics committee prior to participant recruitment, and the procedure was conducted in accordance with the Declaration of Helsinki and the European Union Good Clinical Practice Standards. Participants were recruited through a non-probabilistic convenience sampling, via social networks and including social media platforms such as Facebook, LinkedIn, and Instagram, and researchers’ acquaintances (email and WhatsApp), using snowball sampling strategy. An elaborated online questionnaire was created via Google Forms. Families were provided an informed consent before completing the survey, in which they were apprised that participation was completely anonymous, confidential and voluntary. To protect the confidentiality and anonymity of the data, codes were assigned to identify the participants. Once participants agreed to collaborate, data were collected at three times: 1 month (T1), 7 months (T2) and 1 year and a half after the lockdown (T3). At time 1 (16th April 2020), Spain followed a mandatory confinement, and the schools were closed. At time 2 (20th October 2020), educational centres were opened with some restrictions: wearing masks, social distancing and hygienic measures and a second wave of contagions appeared. At time 3 (25th October 2021), children were at schools and coincided with a less restrictive phase.

### Data analysis

IBM SPSS 27 was used for all statistical analyses. First, frequencies and descriptive analysis were run to examine the level of trait and state anxiety, sleep habit disturbances, executive dysfunction as a general index and its three subscales (emotional dysregulation, cognitive rigidity and disorganisation). These results were provided by calculating means and standard deviations of the total sample in the three times (T1, T2, T3). Also, as we do not have baseline pre-pandemic data, this information was found out through psychometric manuals that provided standardised and normalised data before the appearance of the pandemic. These data were used to merely compare it to the results of the present study. Second, normality, independence and homoscedasticity assumptions were verified to choose the most suitable test to analyse the differences among times (T1, T2, T3). Repeated measures ANOVA and post hoc tests were applied to explore the specific differences of trait and state anxiety, sleep disturbances and executive dysfunction (both as a general index and its three dimensions) among the three times. The effect size of post hoc tests was obtained through the calculation of Cohen’s d to determine the magnitude of differences. The interpretation of the effect size was according to Cohen (1988). Bonferroni’s correction was applied in post hoc analysis. Sphericity assumption was checked, and when it was violated (*p* < 0.05), the Greenhouse–Geisser’s correction was applied. Differences were considered statistically significant when *p* < 0.05.

Also, we ran Pearson correlations among the measured variables: state/trait anxiety, sleep habits and executive functions, and the interpretation of this Hernández-Lalinde et al. (2018) was used. Finally, multiple linear regressions were carried out to create possible models to explain the influence of state/trait anxiety and sleep habit variables as predictors on executive functioning during the COVID-19 pandemic (T1, T2, T3).

## Results

First, before presenting the findings related to differences among the three times of assessment (T1, T2, T3) in trait/state anxiety, sleep habit disturbances and executive dysfunctions, the average scores on the different assessment scales used in this study were compared with the average scores which appeared in the test manuals, understanding the latter group as a normative pre-pandemic population. The data obtained from pre-pandemic samples and the pandemic samples are shown in Table 2.

**Table 2:** Means for state/trait anxiety, sleep habits and executive functioning of pre-pandemic samples and COVID-19-pandemic samples assessed at T1, T2 and T3

	Pre-pandemic samples	T1-pandemic sample	T2-pandemic sample	T3-pandemic sample
State anxiety	31.2	35.0	95.0	64.5
Trait anxiety	35.7	33.6	79.9	82.4
Sleep habits disturbances	7.4	13.2	15.0	14.7
Executive dysfunctions	45.9	41.8	59.4	37.5

**Fig. 1:**
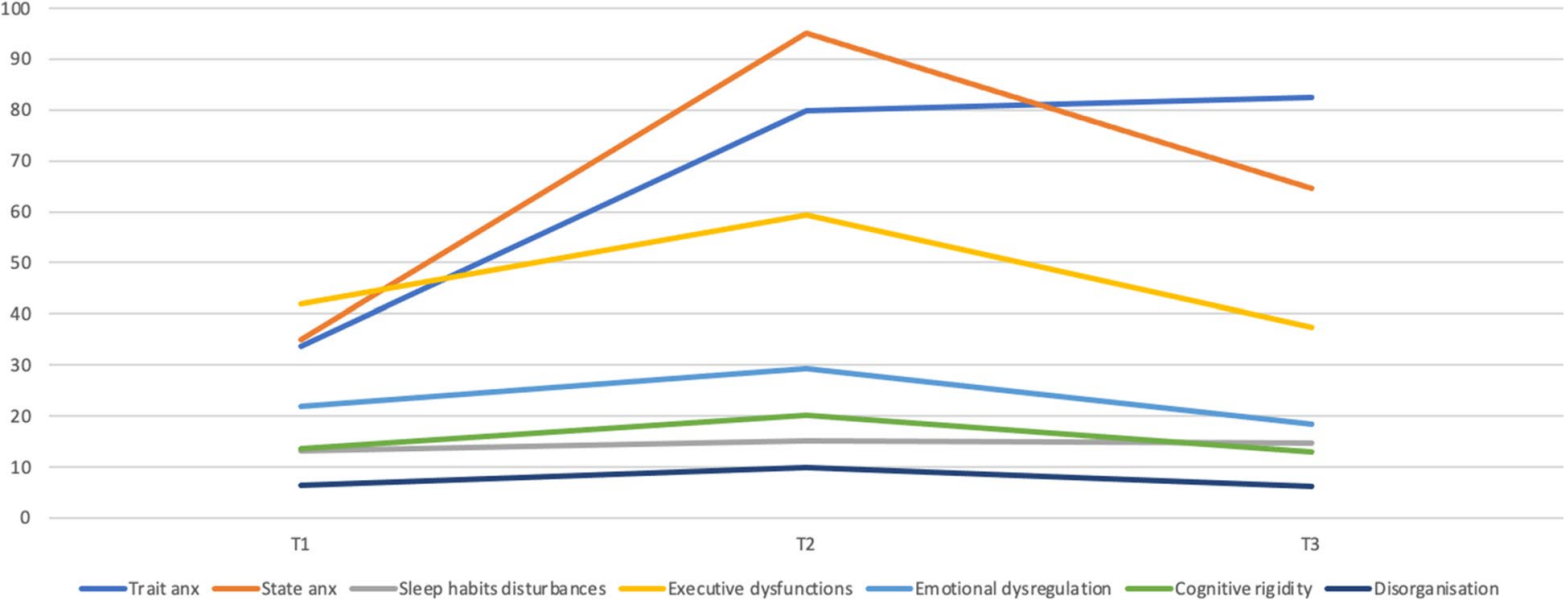
This graph shows the means of the outcome variables (trait/state anxiety, sleep habits disturbances, executive dysfunctioning, emotional dysregulation, cognitive rigidity and disorganisation) during the three times of assessment

Figure 1 presents the level of trait and state anxiety and sleep habit disturbances during 1 year and a half of the COVID-19 pandemic in the three times of assessment (T1, T2, T3). Regarding the trait anxiety, results showed significant differences between T1 (*M* = 33.6) and T2 (*M* = 79.9), t(133) =  − 26.42, *p* < 0.001, *d* =  − 3.33 and T1 and T3 (*M* = 82.4) and t(52) =  − 27.18, *p* < 0.001, *d* =  − 3.72. Although differences between T2 and T3 were not statistically significant, it seemed to have a higher level of trait anxiety in T3. Moreover, these results presented a significant increase of the level of state anxiety from T1 (*M* = 35.0) to T2 (*M* = 95.0), t(133) =  − 12.19, *p* < 0.001, *d* =  − 1.86, but a significant decrease was highlighted from T2 to T3 (*M* = 64.5), t(52) = 6.08, *p* < 0.001, *d* = 0.63. These results also pointed out that the level of sleep habit disturbances was significantly higher in T2 (*M* = 15.0) than in T1 (13.2), t(133) =  − 4.92, *p* < 0.001, *d* =  − 0.67, and also, T3 indicated a higher level of sleep disturbances (*M* = 14.7) than in T1, t(52) =  − 3.89, *p* < 0.001, *d* =  − 0.48 (see Tables S1–S4).

Furthermore, alterations in executive functioning as a general index showed a notable increase in T2, reaching a statistically significant difference between T1 (*M* = 41.8) and T2 (*M* = 59.4), t(133) =  − 4.34, *p* < 0.001, *d* =  − 0.57. However, another significant difference was found between T2 and T3, when youngsters demonstrated a highlighted improvement of executive functioning in the last time (T3: *M* = 37.5), t(52) = 4.92, *p* < 0.001, *d* = 0.58. The same pattern was identified in emotional dysregulation, cognitive rigidity and disorganisation (see Fig. 1, Tables S5–S8).

Regarding the possibility of interpreting results due to the biases of sampling loss, differences in the outcome variables between males and females were run. Table 3 shows that differences are only found in executive functioning at T1.

**Table 3:** Student’s *T*-test. Differences between males and females in trait/state anxiety, sleep habits and executive dysfunctions at T1, T2 and T3

	T1				T2				T3			
	Males M (SD)	Females M (SD)	*t*	*p*	Males M (SD)	Females M (SD)	*t*	*p*	Males M (SD)	Females M (SD)	*t*	*p*
State anxiety	34.62 (8.43)	35.45 (9.03)	− 1.460	0.083	98.08 (23.66)	91.27 (29.09)	1.496	0.137	61.91 (25.12)	68.85 (27.23)	− 0.945	0.349
Trait anxiety	33.52 (7.47)	33.65 (7.51)	− 0.253	0.800	80.66 (9.61)	79.02 (9.92)	0.972	0.333	82.39 (8.76)	82.35 (7.88)	0.018	0.985
Sleep habit disturbances	12.99 (3.56)	13.39 (3.75)	− 1.647	0.100	14.91 (2.58)	15.18 (2.55)	0.535	− 0.623	14.91 (2.79)	14.40 (1.39)	0.757	0.453
Executive dysfunctions	44.35 (16.56)	38.96 (16.27)	5.039	< 0.001	56.74 (18.69)	62.57 (16.91)	0.064	− 1.871	39.42 (16.48)	34.20 (16.65)	1.114	0.270

According to the objective regarding the relationship among trait/state anxiety, sleep habits and executive functions in children and adolescents during the COVID-19 pandemic through the lens of parents, Table S9 shows the Pearson correlations calculated. Thus, moderate-strong, positive and significant correlations were found among trait/state anxiety, alterations in sleep habits and executive dysfunction at T1. For the T2, moderate, positive and significant correlations were pointed out between state anxiety and disturbances in sleep habits and between state anxiety and executive dysfunctions. Also, weak correlations were found between trait anxiety and executive dysfunctions and disturbances in sleep habits and executive dysfunctions. For the T3, strong, positive and significant correlations were demonstrated between state anxiety and executive dysfunctions.

Finally, Table 4 demonstrates the multiple linear regressions which were carried out to examine the influence of trait/state anxiety and sleep habits on executive functions for T1, T2 and T3. State anxiety levels were only a significant predictor of executive functions during T1, T2 and T3. The remaining variables trait anxiety and sleep habits were only significant at T1.

**Table 4:** Regression analysis of trait/state anxiety and sleep habit variables as predictors of executive functions for T1, T2 and T3

Variable	Executive functions (T1)				
	Model 1				
	B	SE	*β*	*p*	95% *CI*
State anxiety	0.734	0.067	0.385	< 0.001	0.602–0.867
Trait anxiety	0.394	0.078	0.178	< 0.001	0.240–0.548
Sleep habits	0.627	0.134	0.138	< 0.001	0.364–0.890
F(3) = 172.986; *p* < 0.001; *R*^2^ = 0.354					
Variable	Executive functions (T2)				
	Model 2				
	B	SE	*β*	*p*	95% *CI*
State anxiety	0.359	0.064	0.523	< 0.001	0.233–0.485
Trait anxiety	0.167	0.166	0.09	0.315	− 0.495–0.161
Sleep habits	0.072	0.584	0.01	0.123	− 1.229–1.084
F(3) = 13.515; *p* < 0.001; *R*^2^ = 0.22					
Variable	Executive functions (T3)				
	Model 3				
	B	SE	*β*	*p*	95% *CI*
State anxiety	0.360	0.082	0.562	< 0.001	0.195–0.524
Trait anxiety	0.190	0.248	0.096	0.447	− 0.308–0.687
Sleep habits	0.113	0.870	0.016	0.897	0.195–0.524
F(3) = 6.993; *p* < 0.001; *R*^2^ = 0.257					

## Discussion

The present study has the main objective of analysing the evolution of some psychological variables in children and adolescents residing in Spain (state and trait anxiety, sleep disturbances, executive functioning-emotional dysregulation, cognitive rigidity and disorganisation), using three time points of reference during 1 year and a half of the COVID-19 pandemic with different levels of restriction, according to parents’ perception.

Regarding levels of trait and state anxiety, the data reveal that at the beginning of the pandemic (T1), it appears that these variables were significantly increased compared to pre-pandemic data (data contrasted with those in published standardised tests), with higher levels of state anxiety than trait anxiety. This could be explained by the extreme movement restrictions of the initial lockdown (Garcia de Avila et al., 2020; Lavigne-Cerván et al., 2021; Ravens-Sieberer et al., 2021; Yeasmin et al., 2020; Zhang et al., 2021). The scientific literature developed during this period (March–April 2020) tends to determine the presence of high levels of anxiety in children and adolescents (El Refay et al., 2021; Levita et al., 2020; Pisano et al., 2020; Racine et al., 2021; Saurabh & Ranjan, 2020; Zhou et al., 2020). However, there is also some research that has found normalised anxiety indexes (Berry et al., 2021; Quero et al., 2021; Wang et al., 2021). For instance, the “COMPASS” study stated that the differences obtained between 2019 and 2020 were lower than the changes observed between 2018 and 2019 (Bélanger et al., 2021).

Seven months later (T2), in October 2020, anxiety levels increased significantly compared to the original confinement, following a similar pattern to T1, with state anxiety being higher than trait anxiety. These moments coincide with the appearance of the second wave of contagions that Spain suffered. Schools and universities opened in face-to-face/blended learning modality but with severe restrictive measures. At T3, 18 months after T1, a change in the levels of state and trait anxiety was detected. Although there was a significant increase in T2 and T3 compared to T1, the most relevant findings were that trait anxiety exceeded state anxiety at T3. These results could suggest that the anxiety prolonged over time has implied a change in the pattern of trait anxiety in the population assessed. Findings consistent with this research suggest that high levels of stress over time can lead to mental health problems in children and adolescents, in turn worsening the mental health of their families (Andres et al., 2022; Brown et al., 2020; and Romero et al., 2020).

Longitudinal research conducted in different parts of the world has studied the evolution of some variables measured in relation to the mental health of children and adolescents from the beginning of the pandemic up to the present (Elharake et al., 2022). Specifically, a longitudinal study conducted in Germany aimed to measure changes in the mental health of children and adolescents. The results were similar to those presented in our study. As for the anxiety variable, it was revealed that it increased at all times when compared to the pre-COVID situation (before COVID-19 pandemic: 15%; time 1: 24%; time 2: 30%; time 3; 27%). Similarly, a systematic review with meta-analysis suggested that longitudinal studies conducted during different phases of the pandemic could corroborate the negative implications on mental health in children and adolescents (Ettman et al., 2022; Racine et al., 2021).

Concerning sleep disturbances, the data showed a similar behaviour to values obtained with the trait anxiety variable. Sleep disturbances detected by parents occurred at all measured time points, especially at T2, with a slight non-significant decrease in T3. This may indicate that the sleep problems detected at the beginning of the pandemic have been worsening over time. Studies conducted from March 2000 to the end of 2021 have revealed sleep quality declines (Cellini et al., 2020), observing such as insomnia (Zhou et al., 2020), nightmares (Scarpelli et al., 2021), sleepwalking (Pérez-Carbonell et al., 2020) and night terrors (Herrero San Martin et al., 2020).

The first study we conducted with the T1 data reported significant positive correlations between anxiety levels and alterations in sleep habits (Lavigne-Cerván et al., 2021). Other authors also agree with our findings, reporting high levels of anxiety with alterations in sleep routines, demonstrating the direct impact of one variable on the other (Becker et al., 2021; Morin et al., 2022). A systematic review compiling studies conducted during the pandemic has shown that 26% of the sample of children and adolescents studied showed symptoms of anxiety, and 44% had sleep disturbances (Miller & Cappuccio, 2021).

Furthermore, the findings found in all measures of executive functioning show the same pattern of behaviour as the state anxiety variable over time. Results seem to show a deterioration in the general index of executive functioning as well as in the scales of emotional regulation, cognitive rigidity/flexibility and planning/organisation. In agreement with these results, other authors highlight that parents perceive a worsening in executive and academic functioning during the COVID-19 (T1) confinement period (Andrés et al., 2022; Hanno et al., 2022; Lavigne-Cervan et al., 2021b, Polizzi et al., 2021). However, when comparing T2 and T3, no significant differences have been found, but some improvement has been observed in the latter period (T3) in relation to the second one (T2). Most of the studies carried out are cross-sectional, and longitudinal studies assessing alterations in the executive system are scarce to date. Only one study conducted in Jordan has been found so far, which took two measurements, one during confinement and another after it. According to this study, a direct effect of the COVID-19 pandemic was observed on executive functioning. Additionally, they found that the organisation subscale was the highest during lockdown, whereas they observed higher scores on the strategic planning scale after the lockdown (Moh'd and S., & Hasan, M, 2021). These results are in line with those obtained in the present study. Most of the longitudinal studies that have been carried out on the COVID-19 situation have focused their research on emotional but not cognitive variables (Elharake et al., 2022).

Also, when monitoring health indicators among children and adolescents, it is important to understand the magnitude of nonresponders and the impact this may have on the prevalence (Cheung et al., 2017). Therefore, in order to provide as much reliable information as possible, bias analysis was carried out. In fact, sociodemographic data at T1, T2 and T3 are pretty similar to each other, and the outcome variables of interest (anxiety, sleep and executive functions) do seem to differ only in executive functions at T1 between males and females. As can be seen, most of the study sample is composed of females, and this could be a reason for a bias estimation. As a matter of a fact, previous studies have shown that females are more prone to response surveys, and controlling this variable could decrease the likelihood of mistakes when interpreting the results (Cheung et al., 2017; Criqui et al., 1987).

According to the results of the present investigation, an initial cross-sectional study by Lavigne-Cerván et al. (2021a) revealed that there were positive correlations between anxiety, sleep disturbances and executive dysfunction. In addition, state anxiety was found to be the variable with the greatest weight in the model that would explain the alteration in executive functioning through a regression analysis.

Pearson’s correlations and the multiple linear regressions data found support the hypothesis that there is a correlation among the measured variables at the three times of assessment during the COVID-19 pandemic. These data suggest that the pandemic appears to be affecting anxiety in children and adolescent population, which in turn may affect sleep habits and negatively impact executive functioning. Although the strength of the correlations is varying during this period of time, moderate-strong correlations between state anxiety and executive dysfunctions were maintained. Psychological reactions to a pandemic appear to be acute, and long-term emotional consequences can be observed (Li et al., 2020).

### Strengths and limitations

In the present study, a strength was to perform a longitudinal study using exclusively the same participants as those who composed the first sample. This difficulty was also one of the main limitations due to the loss of participants, which implies a cautious interpretation of the results. Besides, despite the fact that the initial sample is quite large and includes different regions of the country, it is true that it is adjusted only to the Spanish population. Nevertheless, the national and international studies cited throughout this paper could support the results obtained in the present study at an international level and hence their inference and generalisation. This study may also fill the gap of longitudinal studies in the scientific literature focused on children and youth population, which address the difficulties derived from the pandemic caused by COVID-19 on mental health (especially on anxiety, sleep and executive functioning).

Another strength we have found is that we have been able to explore how all the changes have affected variables as important for child and adolescent development as those related to executive functioning since March 2020. The scientific community has focused on studies of a socio-emotional nature, but not on cognitive variables, such as executive functioning. We believe that it has been relevant to explore the state of certain cognitive variables whose implication in daily routines and decision-making is essential and even more in children and adolescents who are in the midst of their development.

Finally, although the data analysis conducted has allowed us to examine the levels of anxiety, sleep disturbances and executive functioning at different times during the pandemic, it does not provide us with an accurate understanding of the variables that have led to this. Differences among the times measured may be due to unknown variables that have not been considered during the present study. However, it is clear that there are important effects that should be taken into account by the social, health and/or educational services that attend to this population. This would enable them to implement preventive and/or therapeutic programmes-interventions as soon as possible, focussing not only on the mental health of children and adolescents but also that of their parents. In this sense, it would be necessary for institutions to implement programmes and psychological counselling for the entire family nucleus.

## Conclusions and future research

This investigation indicates that the rates of anxiety, sleep habits and problems related to executive functioning in Spanish children and adolescents during the COVID-19 pandemic appear to have deteriorated over time. This contributes to support the idea that the maintenance of a pandemic situation appears to influence the psychological health of the child and adolescent population.

As for the immediate future, monitoring over time of the variables studied and observing their evolution, especially of trait anxiety, are advisable. We are concerned about the maintenance of elevated levels of this variable over time. These findings lead to a new hypothesis that will consist of determining whether these levels, considered pathological before the pandemic, will return to standardised ones established in an unrestricted context.

## Supplementary Information

**Additional file 1**: **Table S1.** Repeated-measures ANOVA analysis. Descriptive analysis and differences among the three measured times in trait and state anxiety, sleep disturbances, executive functioning as a general index, emotional dysregulation, cognitive rigidity and disorganisation in the total sample. **Table S2.** Post-hoc tests in trait anxiety among the three measured times in the total sample. **Table S3.** Post-hoc tests in state anxiety among the three measured times in the total sample. **Table S4.** Post-hoc tests in sleep disturbances among the three measured times in the total sample. **Table S5.** Post-hoc tests in executive functioning as a general index among the three measured times in the total sample. **Table S6.** Post-hoc tests in emotional dysregulation among the three measured times in the total sample. **Table S7.** Post-hoc tests in cognitive rigidity among the three measured times in the total sample. **Table S8.** Post-hoc tests in disorganisation among the three measured times in the total sample. **Table S9.** Pearson correlations among trait/state anxiety, sleep habits and executive functions in children and adolescents during the COVID-19 pandemic through the parents’ perception.

## Abbreviations

WHO: World Health Organization
T: Time
STAIC: State-Trait Anxiety Inventory for Children
STAI: State-Trait Anxiety Inventory
BEARS: Sleep-screening tool for sleep disturbances in childhood
BRIEF-2: Behavioral Evaluation of Executive Function-2
