# Supplementary material for: Anxiety, sleep habits and executive function during the COVID-19 pandemic through parents’ perception: a longitudinal study
Source: Psicol Reflex Crit. 2023 Mar 29;36:8. doi: 10.1186/s41155-023-00251-5 (PMC10050808; doi:10.1186/s41155-023-00251-5)
Supplement: Supplementary file 1 — Additional file 1: Table S1. Repeated-measures ANOVA analysis. Descriptive analysis and differences among the three measured times in trait and state anxiety, sleep disturbances, executive functioning as a general index, emotional dysregulation, cognitive rigidity and disorganisation in the total sample. Table S2. Post-hoc tests in trait anxiety among the three measured times in the total sample. Table S3. Post-hoc tests in state anxiety among the three measured times in the total sample. Table S4. Post-hoc tests in sleep disturbances among the three measured times in the total sample. Table S5. Post-hoc tests in executive functioning as a general index among the three measured times in the total sample. Table S6. Post-hoc tests in emotional dysregulation among the three measured times in the total sample. Table S7. Post-hoc tests in cognitive rigidity among the three measured times in the total sample. Table S8. Post-hoc tests in disorganisation among the three measured times in the total sample. Table S9. Pearson correlations among trait/state anxiety, sleep habits and executive functions in children and adolescents during the COVID-19 pandemic through the parents’ perception. [file 41155_2023_251_MOESM1_ESM.docx]

Table S1. Repeated-measures ANOVA analysis. Descriptive analysis and differences among the three measured times in trait and state anxiety, sleep disturbances, executive functioning as a general index, emotional dysregulation, cognitive rigidity and disorganisation in the total sample.

|  | M (SD) | | | F | p | η² / ε | ω² |
| --- | --- | --- | --- | --- | --- | --- | --- |
|  | T1 | T2 | T3 |  |  |  |  |
| Trait anxietyª | 33.6 (7.49) | 79.9 (9.74) | 82.4 (8.36) | 479.1 | 0.034 | 0.889 | - |
| State anxietyª | 35.0 (8.72) | 95.0 (26.40) | 64.5 (25.90) | 74.3 | <0.001 | 0.588 | 0.548 |
| Sleep disturbances | 13.2 (3.65) | 15.0 (2.56) | 14.7 (2.37) | 13.5 | <0.001 | 0.206 | 0.087 |
| Executive dysfunctionª | 41.8 (16.63) | 59.4 (18.08) | 37.5 (16.58) | 14.47 | <0.001 | 0.218 | 0.186 |
| Emotional dysregulationª | 21.9 (10.01) | 29.3 (10.50) | 18.4 (9.74) | 8.81 | 0.002 | 0.145 | 0.115 |
| Cognitive rigidityª | 13.5 (6.55) | 20.05 (6.65) | 12.9 (6.57) | 11.67 | <0.001 | 0.183 | 0.151 |
| Disorganizationª | 6.5  (3.17) | 10.0 (3.21) | 6.1 (3.26) | 16.78 | <0.001 | 0.244 | 0.199 |

ªThe assumption of sphericity was violated (p < 0.05). Greenhouse-Geisser’s correction was applied.

Table S2. Post-hoc tests in trait anxiety among the three measured times in the total sample.

| Times comparisons | | t | p | d |
| --- | --- | --- | --- | --- |
| 1 | 2 | -26.42 | <0.001 | -3.33 |
| 1 | 3 | -27.18 | <0.001 | -3.72 |
| 2 | 3 | -0.76 | 0.447 | -0.13 |

Table S3. Post-hoc tests in state anxiety among the three measured times in the total sample.

| Times comparisons | | t | p | d |
| --- | --- | --- | --- | --- |
| 1 | 2 | -12.94 | <0.001 | -1.86 |
| 1 | 3 | -10.56 | <0.001 | -1.45 |
| 2 | 3 | 4.56 | <0.001 | 0.63 |

Table S4. Post-hoc tests in sleep disturbances among the three measured times in the total sample.

| Times comparisons | | t | p | d |
| --- | --- | --- | --- | --- |
| 1 | 2 | -4.92 | <0.001 | -0.67 |
| 1 | 3 | -3.89 | <0.001 | -0.48 |
| 2 | 3 | 1.02 | 0.307 | 0.17 |

Table S5. Post-hoc tests in executive functioning as a general index among the three measured times in the total sample.

| Times comparisons | | t | p | d |
| --- | --- | --- | --- | --- |
| 1 | 2 | -4.34 | <0.001 | -0.57 |
| 1 | 3 | 0.58 | 0.199 | 0.18 |
| 2 | 3 | 4.92 | <0.001 | 0.58 |

Table S6. Post-hoc tests in emotional dysregulation among the three measured times in the total sample.

| Times comparisons | | t | p | d |
| --- | --- | --- | --- | --- |
| 1 | 2 | -3.11 | 0.005 | -0.45 |
| 1 | 3 | 0.88 | 0.06 | 0.26 |
| 2 | 3 | 3.99 | <0.001 | 0.47 |

Table S7. Post-hoc tests in cognitive rigidity among the three measured times in the total sample.

| Times comparisons | | t | p | d |
| --- | --- | --- | --- | --- |
| 1 | 2 | -4.22 | <0.001 | -0.55 |
| 1 | 3 | -0.09 | 0.932 | -0.02 |
| 2 | 3 | 4.14 | <0.001 | 0.50 |

Table S8. Post-hoc tests in disorganisation among the three measured times in the total sample.

| Times comparisons | | t | p | d |
| --- | --- | --- | --- | --- |
| 1 | 2 | -4.77 | <0.001 | 0.65 |
| 1 | 3 | 0.46 | 0.645 | 0.09 |
| 2 | 3 | 5.23 | <0.001 | 0.62 |

Table S9. Pearson correlations among trait/state anxiety, sleep habits and executive functions in children and adolescents during the COVID-19 pandemic through the parents’ perception.

|  | 1. | 2. | 3. | 4. | 5. | 6. | 7. | 8. | 9. | 10. | 11. | 12. |
| --- | --- | --- | --- | --- | --- | --- | --- | --- | --- | --- | --- | --- |
| 1. State anxiety (T1) | - |  |  |  |  |  |  |  |  |  |  |  |
| 2. Trait anxiety (T1) | 0.654*** | - |  |  |  |  |  |  |  |  |  |  |
| 3. Sleep habits disturbances (T1) | 0.422*** | 0.423*** | - |  |  |  |  |  |  |  |  |  |
| 4. Executive dysfunction (T1) | 0.559*** | 0.488*** | 0.375*** | - |  |  |  |  |  |  |  |  |
| 5. State anxiety (T2) |  |  |  |  | - |  |  |  |  |  |  |  |
| 6. Trait anxiety (T2) |  |  |  |  | 0.508*** | - |  |  |  |  |  |  |
| 7. Sleep habits disturbances (T2) |  |  |  |  | 0.371*** | 0.268*** | - |  |  |  |  |  |
| 8. Executive dysfunction (T2) |  |  |  |  | 0.481*** | 0.178* | 0.180* | - |  |  |  |  |
| 9. State anxiety (T3) |  |  |  |  |  |  |  |  | - |  |  |  |
| 10. Trait anxiety (T3) |  |  |  |  |  |  |  |  | 0.280* | - |  |  |
| 11. Sleep habits disturbances (T3) |  |  |  |  |  |  |  |  | 0.256 | 0.139 | - |  |
| 12. Executive dysfunction (T3) |  |  |  |  |  |  |  |  | 0.531*** | 0.159 | 0.122 | - |

*p<0.05; **p<0.01; ***p<0.001
